# Motor Cortex Reorganization in Limb Amputation: A Systematic Review of TMS Motor Mapping Studies

**DOI:** 10.3389/fnins.2020.00314

**Published:** 2020-04-21

**Authors:** Muhammed Enes Gunduz, Camila Bonin Pinto, Faddi Ghassan Saleh Velez, Dante Duarte, Kevin Pacheco-Barrios, Fernanda Lopes, Felipe Fregni

**Affiliations:** ^1^Laboratory of Neuromodulation & Center for Clinical Research Learning, Department of Physical Medicine and Rehabilitation, Harvard Medical School, Spaulding Rehabilitation Hospital, Boston, MA, United States; ^2^Unidad de Investigación Para la Generación y Síntesis de Evidencias en Salud, Universidad San Ignacio de Loyola, Lima, Peru

**Keywords:** amputation, phantom limb pain, transcranial magnetic stimulation, motor cortex reorganization, cortical mapping

## Abstract

**Purpose:** The purpose of this systematic review is to evaluate motor cortex reorganization in amputees as indexed by transcranial magnetic stimulation (TMS) cortical mapping and its relationship with phantom limb pain (PLP).

**Methods:** Pubmed database were systematically searched. Three independent researchers screened the relevant articles, and the data of motor output maps, including the number of effective stimulation sites, center of gravity (CoG) shift, and their clinical correlations were extracted. We calculated a pooled CoG shift for motor cortex TMS mapping.

**Results:** The search yielded 468 articles, 11 were included. Three studies performed correlation between the cortical changes and PLP intensity, and only one study compared cortical mapping changes between amputees with pain and without pain. Results showed (i) enlarged excitable area and a shift of CoG of neighboring areas toward the deafferented limb area; (ii) no correlation between motor cortex reorganization and level of pain and (iii) greater cortical reorganization in patients with PLP compared to amputation without pain.

**Conclusion:** Our review supports the evidence for cortical reorganization in the affected hemisphere following an amputation. The motor cortex reorganization could be a potential clinical target for prevention and treatment response of PLP.

## Introduction

Amputation leads to reorganization in the motor cortex. Several neurophysiological and neuroimaging studies pointed out that there is cortical reorganization associated with limb amputation (Schwenkreis et al., 2003) and that one of the consequences of reorganization is phantom limb pain (PLP) (Flor et al., 1995, 2001, 2006; Foell et al., 2014). Increasing evidence suggests that changes in the primary motor cortex are observed in amputees with PLP (Schwenkreis et al., 2001; Mercier and Léonard, 2011). Despite PLP having a high incidence, affecting up to 85% of the amputees (Sherman et al., 1980; Pezzin et al., 2000), PLP underlying mechanism remains controversial and unclear. One hypothesis is that the lack of inhibitory activity in the sensory-cortical feedback pathways leads to continued efferent motor cortical commands due to enhanced cortical excitability (Ziemann et al., 1998; Zagha et al., 2016; Ruddy et al., 2018). Therefore, cortical deafferentation and lack of inhibitory activity may play a role in phantom pain that still needs to be elucidated. This notion is also supported by studies showing decreased intracortical inhibition in neuropathic pain and other chronic pain syndromes (Castillo Saavedra et al., 2014; Tarrago Mda et al., 2016).

In this context, two techniques -transcranial magnetic stimulation (TMS) and magnetic resonance imaging (MRI)- have been used to assess cortical reorganization following an amputation and to elucidate pathophysiologic mechanisms of PLP, as well as provide clues to optimize the rehabilitation of individuals with PLP in the clinical context. TMS is used to brain mapping of the cortical regions (Wagner et al., 2007; Dayan et al., 2013; Rossini et al., 2015). For motor cortex mapping, by applying TMS to different locations in the scalp referenced by vertex, MEP amplitudes can be evoked in target muscles (Thickbroom et al., 1999). Then the map of the area is created by the MEP responses collected at contralateral muscle. The hot spot, center of gravity (CoG), and number of effective stimulation site are the main parameters evaluated. While hot spot represents the maximum value of the MEP response, the CoG is spatial average optimal site (Rossini et al., 2015). The number of effective sites represent the surface area of the muscle representation. In amputees, these can be especially useful in the characterization of cortical reorganization. The stability of TMS measures supports use of TMS to assess underlying cortical plasticity in amputees (Hetu et al., 2011).

TMS studies provide evidence of motor cortex excitability changes. Cohen et al. (1991) was the first to describe the motor cortex reorganization following an amputation, showing larger motor evoked potentials (MEP), and increased number of excitable stimulation sites for the muscles immediately proximal of the stump. Similarly, Pascual-Leone et al. (1996) performed TMS cortical mapping before and after upper limb amputation and showed cortical reorganization as the neighboring areas “invade” the deafferented zone (enlargement and/or shift of the targeted muscle motor area) in amputees (Pascual-Leone et al., 1996). These results further supported the evidence of motor output map alterations in amputees. However, Gagné et al. (2011) challenged these findings by showing no significant difference in the map areas or the shift of their locations in traumatic upper limb amputees (Gagné et al., 2011).

There is no clear understanding as to why and how amputation and subsequent cortical reorganization relates to pain as some TMS studies showed no correlation between pain intensity and shift in cortical map (Irlbacher et al., 2002; Schwenkreis et al., 2003). Even though changes in the cortical representation of neighboring areas are frequently observed (Flor et al., 1995; Lotze et al., 2001), the same mixed results are observed in fMRI studies. Lotze et al. (2001) show that upper limb amputees with PLP have a shift of the lip area into the deafferented hand motor area and that the shift is positively correlated with the PLP intensity. These results are also replicated in 5 fMRI studies (Lotze et al., 1999; MacIver et al., 2008; Diers et al., 2010; Foell et al., 2014; Raffin et al., 2016). However, although Makin et al. (2013) and Kikkert et al. (2018) also found a correlation between the level of reorganization and PLP, this correlation was negative indicating that the preserved structural and functional organization in the brain of the amputated limb area was related to more pain (Makin et al., 2013; Kikkert et al., 2018).

The TMS measurements of motor cortex can further help to clarify the discrepancy on cortical reorganization findings following an amputation and to define which are the neural correlates of phantom limb pain. Therefore, we conducted this review in studies including patients with lower or upper limb amputation that have been assessed by TMS cortical mapping as to determine (i) whether there is a shift in the center of gravity of the cortical mapping when combining data from these studies; (ii) whether this shift is associated with pain and (iii) whether there is a difference between amputation without pain and amputation with PLP.

## Methods

### Literature Search

A systematic search was conducted in the PubMed database, utilizing the following keywords: “transcranial magnetic stimulation” or “TMS” or “cortical reorganization” and “amputees” or “amputation” and/or “phantom limb pain.” The last search update was run in December 2018. No additional filters (e.g., publication year) were set. A manual search was also conducted to find other potential articles based on references identified in the individual articles.

### Literature Selection: Inclusion and Exclusion Criteria

We included all original articles and case reports that reported the assessment of cortical reorganization in amputees using TMS. Only articles written in English were included. We, therefore, excluded the following articles: (1) animal studies; (2) review articles; (3) letters to the editor; (4) editorials and (5) duplicate studies.

Duplicated records were removed and three reviewers (CBP, FGSV, FL) screened all titles and abstracts following the pre-specified framework and selection criteria. Discrepancies were solved by another reviewer independently (MEG). After the title and abstract selection, full text of selected reports was sought and analyzed discrepancies were solved by consensus between all authors.

### Quality Assessment

To assess the quality of the included TMS studies, we used the checklist developed by Chipchase et al. (2012) following the standard procedure describe by the authors. This tool the factors that should be reported and/or controlled in TMS studies. We assigned a value of zero if they do not report and justify the criteria in the manuscript and one if they do so. Then, we calculate the total score per study, a higher number correspond high quality. The quality evaluation was assessed by two reviewers (MEG and KP-B), and discrepancies were solved by a third reviewer independently (FF).

### Data Extraction

After a detailed review of the articles, the authors identified and collected the most significant parameters measured during the evaluation of cortical reorganization by TMS. Data were collected and reviewed by two authors independently (MEG and KP-B). The following list of variables was structured in order to extract the proper evaluation of cortical reorganization in amputees, when available:

a) TMS Evidence of Cortical Reorganization: Cortical Reorganization measured by Motor Output Maps including (i) number of effective stimulation sites and (ii) center of gravity (CoG) shift.

b) Clinical Correlations: Illustration of how previous parameters correlate with clinical characteristics (negative and positive correlations), including phantom limb pain, residual pain, telescoping, use of prosthesis, time since amputation, and level of amputation.

### Pooled CoG Calculation

The formula used for data extraction, documented the Cz referenced medial-lateral coordinates in mm (x axis) ± SD, and posterior-anterior coordinates in mm (y axis) ± SD. The studies were divided according to the amputated limb (lower vs. upper), and according target muscle in the TMS protocol (upper-limb muscles, lower-limbs muscles, or face muscles), in order to avoid anatomical heterogeneity in analysis. Then we calculated the weighted arithmetical mean with the x and y values and the SD pooled, following the Cohen's effect size formula (Thalheimer and Cook, 2002; Durlak, 2009). We represented the pooled CoG in a cartesian plot and 3D brain template consider Cz as a reference. The data were processed using MATLAB R2018a software.

### Management of Missing Data

In the case of unreported, missing, or unclear data regarding the primary outcome data (i.e., CoG) the authors were contacted. Besides that, we used Web Plot Digitizer v.3.11 to extract data from relevant graphs. If authors were unresponsive or extracting the data graphically was not possible, the study was excluded from the quantitative analysis.

## Results

### Studies Retrieval

**Figure 1:**
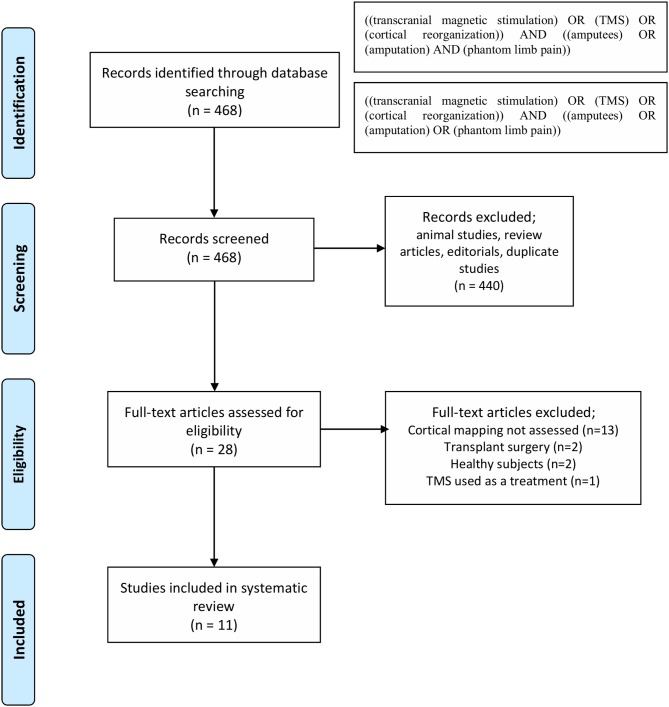
Literature search flow-chart.

The results of search strategy summarized in Figure 1 as PRISMA statement flow diagram (Moher et al., 2009). The literature search resulted in 468 articles. Based on titles and abstracts screening, 440 articles were excluded. Then, the 28 remaining articles were screened by reading the full text for cortical reorganization measurements using TMS. In this phase, 17 excluded as they did not report measures cortical reorganizations and therefore, 11 articles were included.

### Demographic Findings

Table 1 indicates the demographic and clinical information of the sample included in the studies analyzed here. Ten studies were on upper limb amputees, only 1 study evaluated lower limb amputees. In the selected articles, the aggregate number of participants was 84 (77 upper limb amputees and seven lower limb amputees). Most participants were adults, and the average age was 36.3 (range of 14–78). The majority of participants was male, and gender distribution was 77 males and seven females. Time since amputation wasn't an inclusion criterion for the included articles, and it varied from 1 month to 52 years. From the 11 articles included, only seven studies reported if participants suffered or not from PLP; 32 participants (%38.1 of total) had PLP. In 4 studies that reported the level of PLP, the intensity was moderate (mean 4.3 on 0–10 scale; 0 no pain, ten worst pain imaginable).

**Table 1:** Characteristics of included studies.

**References**	**Type of study**	**Sample size**	**Type of amputation**	**Gender**	**Time since amputation (mean ± SD)**	**Phantom limb pain**	**PLP intensity on 0–10 scale (mean ± SD)**	**Type of control (comparison)**	**Number of effective stimulation site**	**CoG shift**	**Clinical correlation**
Schwenkreis et al. (2003)	Cross-sectional	7	Lower limb, all traumatic	6 M/1 F	129.0 ± 122.2 months	All with PLP	5.2 ± 1.8	Interhemispheric and between groups (healthy control)	Not assessed	Significant medial shift of CoG on AF	No correlation
Irlbacher et al. (2002)	Cross-sectional	10	Upper limb, all traumatic	10 M	35 ± 15 years	6 with PLP	4.5 ± 4.3	Interhemispheric and between groups (healthy control)	Increased in affected side	Significant lateral shift on AF	No correlation
Karl et al. (2001)	Cross-sectional study	10	Upper limb, 5 with PLP, 5 without PLP	9 M/1 F	With PLP: 32 ± 7.4 Without PLP: 22.6 ± 18.3 years	5 with PLP	Not reported	Interhemispheric comparison	Increased in affected side	Significant medial shift of target muscles on affected side only in patients with PLP	Not assessed
Schwenkreis et al. (2001)	Cross-sectional study	11	Upper limb, 10 traumatic, 1 cancer	10 M/1 F	122.6 ± 187.8 months	6 with PLP	3.1 ± 3.4	Interhemispheric and between groups (healthy control)	Increased in affected side	Significant lateral shift on affected side	No correlation
Cohen et al. (1991)	Cross-sectional study	8	Upper limb, 7 acquired, 1 congenital	3 M/5 F	1–12 years	Not reported	Not reported	Interhemispheric and between groups (healthy control)	Increased in affected side	Not assessed	Not assessed
Gagné et al. (2011)	Cross-sectional study	8	Upper limb, all traumatic	7 M/1 F	11.6 ± 17.4 years	5 amputees with PLP	4.4 ± 1.8	Interhemispheric comparison	Not assessed	No significant difference	Not assessed
Hamzei et al. (2001)	Case series	7	Upper limb in early childhood, 6 acquired, 1 congenital	7 M	3–38 years	No amputee with PLP	N/A	Interhemispheric comparison	Increased in affected side	2 significant lateral shifts 1 significant medial shift	Not assessed
Kew et al. (1994)	Cross-sectional study	6	Upper limb, 3 traumatic, 3 congenital	5 M/1 F	2–12 years	3 with PLP (traumatic) 3 without PLP (congenital)	Not reported	Interhemispheric comparison and comparison between groups (healthy control)	Traumatic: Increased in affected side Congenital: No difference	Not assessed	Not assessed
Röricht et al. (1999)	Cross-sectional study	15	Upper limb	14 M/1 F	38.7 ± 17.5 years	Not reported	Not reported	Interhemispheric and between groups (healthy control)	8/15 increased in affected side (only 3 significant)	Not assessed	Not assessed
Dettmers et al. (1999)	Case report	1	Upper limb, traumatic	1 M	14 years	No PLP	N/A	Interhemispheric comparison	Increased in affected side	Significant lateral shift on AF	Not assessed
Pascual-Leone et al. (1996)	Case report	1	Upper limb, traumatic	1 M	5 weeks, 4 and 11 months	Not reported	Not reported	Interhemispheric comparison	Not assessed	Invasion of the deafferented area by the face and hand	Not assessed

*AF, Affected hemisphere; CoG, Center of gravity*.

### Study Design and Technical Aspects

Table 2 summarizes the neurophysiological parameters used in the studies included. All included studies used TMS with figure-of-eight-coil to perform cortical mapping. For surface EMG recording, the most frequent muscles evaluated for the case of the affected side in upper limb amputees were: (1) Biceps brachialis (8 papers); (2) deltoid (6 articles); (3) thenar eminence muscles (abductor pollicis brevis, oppones pollicis (1 article); and (4) flexor carpi radialis (1 article). For the case of studies that involved the evaluation of lower limb amputees, the only muscle evaluated was the quadriceps femoris. Also, a couple of studies utilized muscles in face area as controls (zygomaticus and depressor labi inferioris). In most of the cases, the homologous muscle of the healthy side was also evaluated.

**Table 2:** Neurophysiological parameters of included studies.

**References**	**Type of coil**	**Area of interest**	**Muscles evaluated**	**Task performed**	**Size of grid**	**Number of pulses applied**	**Use of prothesis**
Schwenkreis et al. (2003)	Figure-of-eight coil	M1	Quadriceps femoris muscle	No task performed	Until no further MEP could be elicited in steps of 1 cm	8	All patients
Irlbacher et al. (2002)	Figure-of-eight coil	M1	Biceps brachii and first dorsal interosseus	No task performed	1 × 2 cm	5	No prosthesis within 4 years
Karl et al. (2001)	Figure-of-eight coil	M1 and S1	Biceps brachii, Zygomaticus, Depressor labii inferioris	No task performed	7 × 9 cm	3	Not reported
Schwenkreis et al. (2001)	Figure-of-eight coil	M1 and S1	Biceps brachii and deltoid muscles	No task performed	Until no further MEP could be elicited in steps of 1 cm	8	5 Myoelectric; 3 Cosmetic; 3 without
Cohen et al. (1991)	Figure-of-eight coil	M1	Biceps brachii and deltoid muscles	No task performed	1 × 2.5 cm	At least 3	Not reported
Gagné et al. (2011)	Figure-of-eight coil	M1	Biceps brachii and deltoid muscles	3 conditions tested; (1) at rest; (2) during a slight voluntary contraction; (3) during a phantom movement	5 × 3 cm	4	Only 4 patients
Hamzei et al. (2001)	Figure-of-eight coil	M1	Deltoid muscle	no task performed	Until no further MEP could be elicited in steps of 1 cm	Not reported	2 Static prosthesis; 1 Bio-prosthesis; 4 without prosthesis
Kew et al. (1994)	Figure-of-eight coil	M1 and S1	Deltoid, flexor carpi radialis and biceps brachii muscles	No task performed	1 × 2 cm	3	1 Bodypowered; 1 Cosmetic; 3 Myoelectric; 1 Acosmetic
Röricht et al. (1999)	Figure-of-eight coil	M1	Biceps brachii, deltoid and trapezoid muscles	No task performed	1 × 2 cm	5	All patients
Dettmers et al. (1999)	Figure-of-eight coil	M1	Deltoid muscle	No task performed	Until no further MEP could be elicited in steps of 1 cm	Not reported	Not reported
Pascual-Leone et al. (1996)	Figure-of-eight coil	M1 and S1	Right lower facial muscles, thenar and biceps muscles	No task performed	1 × 1 cm	15	Not reported

*M1, The Primary Motor Cortex; S1, The Primary Somatosensory Cortex; MEP, Motor evoked potential*.

Several sizes of grids were observed in the mapping protocol of either upper or lower limb areas. The distance between points in the grid varied from 1 up to 2 cm and the size areas ranged from 1 × 1 cm up to 7 × 9 cm. Another parameter is the number of pulses applied over each intersection of the grid. Numerous variations were observed, finding studies that applied only 3 stimuli per position while others applied either 4, 5, 8, or 15 stimulus per intersection.

### Quality Assessment Results

We evaluated the quality of the 11 included studies, the range of points was from 13 to 20 (out of 26 possible applicable domains). They mostly did not report adequately the following domains: “Coil location and stability (with or without a neuronavigation system)” (90%), “Pulse shape” (90%) “Subjects prescribed medication” (77%), “Use of CNS active drugs (e.g., anti-convulsant)” (77%), “Any medical conditions” (77%), “Amount of relaxation/contraction of target muscles” (77%), and “The time between MEP trials” (72.7%). All studies did not report the “Prior motor activity of the muscle to be tested,” “History of specific repetitive motor activity,” “level of relaxation of muscles other than those being tested,” “Subject attention (level of arousal) during testing” (See Supplementary Table 1).

### Cortical Mapping—Whether There Is a Shift in the Center of Gravity of the Cortical Mapping in Subjects With Limb Amputation

**Figure 2:**
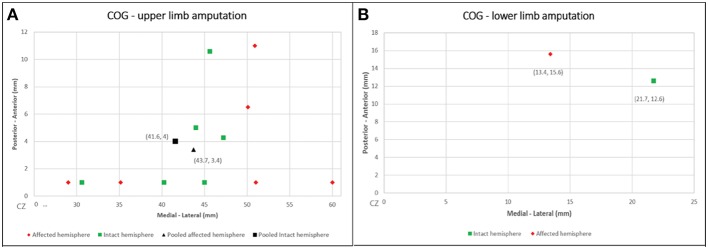
Cartesian plots presenting the CoG coordinates from the included studies and the pooled calculation. **(A)** Upper-limb amputation; **(B)** lower-limb amputation.

**Figure 3:**
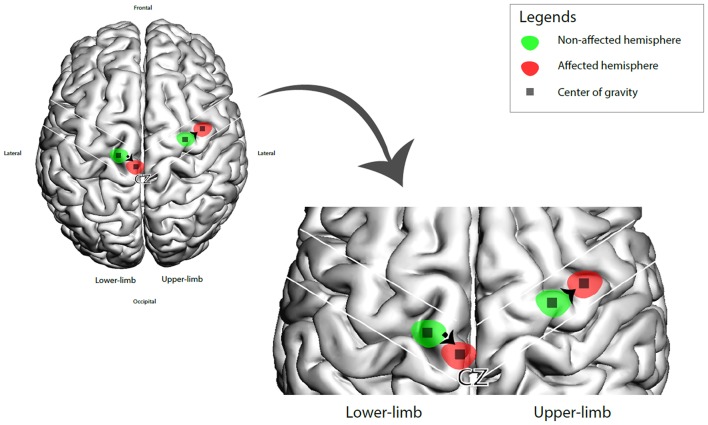
3D brain representation of the CoG shift (affected vs. intact hemisphere) in upper and lower amputee patients.

The CoG coordinates extracted are summarize in Figure 2. We calculated the CoG coordinates of upper-limb amputee patients from 6 studies (*n* = 42) (Dettmers et al., 1999; Hamzei et al., 2001; Karl et al., 2001; Schwenkreis et al., 2001; Irlbacher et al., 2002; Gagné et al., 2011), all of them used upper-limb muscles as TMS targets (biceps brachii, deltoid and trapezoid muscles). We showed a significant difference of CoG between the intact vs. affected hemisphere. The pooled CoG coordinates from the affected hemisphere (contralateral to amputation) were 43.7 ± 8.2 mm (medial-lateral) and 3.4 ± 4.4 mm (posterior-anterior), and from the intact hemisphere were 41.6 ± 7.1 mm (medial-lateral) and 4 ± 4.5 mm (posterior-anterior). We found one study (*n* = 7) (Schwenkreis et al., 2003) on lower-limb amputee patients (the target muscle was quadriceps femoris), the CoG coordinates the affected hemisphere were 13.4 ± 1.5 mm (medial-lateral) and 15.6 ± 5.3 mm (posterior-anterior), and from the intact hemisphere were 21.7 ± 4.3 mm (medial-lateral) and 12.6 ± 6.5 mm (posterior-anterior) (see Figure 3).

Eight studies assessed the number of effective stimulation sites; six showed significant increase in affected hemisphere, one study compared traumatic amputees with congenital amputees and showed increase only in traumatic subjects. One study with 15 subjects evaluated separately each subject and showed increased in affected hemisphere in eight patients while only three were significant (see Table 1).

### Clinical Correlation—Whether This Shift Is Associated With Pain

TMS parameters of cortical reorganization were not correlated with clinical characteristics of enrolled subjects. We found three articles in which investigators attempted to investigate possible correlations (Table 1) (Schwenkreis et al., 2001, 2003; Irlbacher et al., 2002). These studies evaluated age, time since amputation, phantom and stump pain intensity, and their correlation with CoG and number of effective stimulation sites. None of these studies reported any significant correlation between neurophysiologic TMS parameters and clinical features.

### Whether There Is a Difference Between Amputation Without Pain and Amputation With PLP

Additionally, one study compared the CoG between groups with PLP and without PLP (Karl et al., 2001). They specifically reported that the target muscles' CoG were significantly more medial (toward the missing hand area) only in patients with PLP.

## Discussion

In this review, we evaluated cortical mapping reorganization in upper and lower limb amputees and how this correlates with clinical parameters such as phantom limb pain. We showed a pooled lateral mapping shifting (2.1 mm) in upper limp amputees and a medial shift in lower limb (8.3 mm). Besides that, the functional cortical representation of the missing limb was larger and more widespread than the non-affected one. However, most of the articles either did not investigate this change with PLP or those that did show no correlation between the changes in mapping and intensity of PLP.

There is a notion that changes in cortical mapping are related to the presence of acute and chronic pain. One of the ideas recently hypothesized is that pain can itself cause reorganization of the motor cortex (Pinto et al., 2016). The exact mechanisms by which these alterations can correlate with pain presence are far from being understood. Recently, several patterns of changes in the motor cortex and somatosensory cortex have been observed in patients that experience PLP such as (i) Invasion of neighboring areas into the zone of the deafferented limb area, CoG shift, and enlargement of the excitable area (Pascual-Leone et al., 1996); (ii) Clinical correlation between motor cortex reorganization and PLP, and whether cortical mapping changes would be a biomarker for the PLP vs. amputation with no pain; besides the previously mentioned, there are as well two contrasting findings of how these changes are associated with PLP (Karl et al., 2001); (iii) The intensity of pain is associated with the level of reorganization (maladaptive or abnormal/enlargement) (Lotze et al., 2001), or it is associated with the level of preservation of the limb representation (Makin et al., 2013). As to understand these critical patterns, we address each of them separately.

### Invasion of Neighboring Areas Into the Zone of the Deafferented Limb Area; CoG Shift and Enlargement of the Excitable Area

Several research groups have shown that in human subjects, deafferentation of a limb leads to changes in the activity of contralateral cortical areas to the side of amputation (Cohen et al., 1991; Kew et al., 1994). In particular, there is an invasion of the neighboring areas into the deafferented space that corresponds to the missing limb. For example, fMRI studies showed that upper limb amputees that experience PLP have a medial shift in the sensorimotor representation of the lip area into the former hand area, these patients also had an enlarged representation of the lip area during lip movement when compared with amputees with no pain and healthy controls (Lotze et al., 2001). These results were also observed in this review, since following an amputation most of the studies also revealed evidence of cortical reorganization in the affected motor cortex (contralateral to the amputated side) for both lower and upper amputees. The findings of included studies showed displacements of the pooled CoG corresponding to the evaluated muscle (shift in neighboring areas), however, there was no consensus on the direction of the displacement as some authors showed lateralization of the muscle representation (Dettmers et al., 1999; Schwenkreis et al., 2001; Irlbacher et al., 2002) and others showed medial displacement (Pascual-Leone et al., 1996; Karl et al., 2001; Schwenkreis et al., 2003). In our review, one study investigated cortical organization before and after amputation with an single subject, and showed an invasion of the hand deafferented area by the face area (Pascual-Leone et al., 1996). The idea of deafferented zones, is also supported by observations of patients referring sensations in phantoms that are produced after stimulation of the adjacent areas as well as distant areas from the missing limb (Ramachandran et al., 1992). Similar findings were seen when changes of mapping were assessed by TMS, as studies most frequently showed an increase in the number of scalp stimulation sites for the most proximal muscles to the stump, suggesting an enlargement of the cortical representation. The above mentioned provides an insight into the potential underlying changes in cortical sensory-motor representations seen in association with pain in this population.

### Clinical Correlation Between Motor Cortex Reorganization and PLP, and Whether Cortical Mapping Changes Would Be a Biomarker for the PLP vs. Amputation With no Pain

In our review, although none of TMS studies found a significant correlation between cortical mapping and intensity of PLP, as well as stump pain, almost all the analyzed manuscripts showed evidence of cortical reorganization. Considering the cortical reorganization findings seen in patients with PLP (Karl et al., 2001), our results suggest a dissociation between CoG shift and intensity of pain. Different from TMS studies, EEG and fMRI studies, reported correlations between the amount of cortical reorganization and the magnitude of PLP (Flor et al., 1995; Birbaumer et al., 1997; Grusser et al., 2001). Thus, rather than focusing on correlation with the intensity of the pain, future studies should focus on using TMS measurements as neurophysiologic predictors for the identification of potential patients with increased risk in developing phantom pain. Likewise, TMS evaluation can also be used as a follow-up measurement that will allow determining if a specific treatment is leading to plastic changes (in plain words the disorganization is being reorganized) and if these changes can be dependent on the treatment being tested (Pinto et al., 2016).

In regards of the relationship of cortical reorganization with the presence of PLP, only one TMS study compared amputees with and without PLP, showing a significant medial shift in the CoG (toward the missing hand area) only in upper limb amputees with PLP (Karl et al., 2001).

This study by Karl et al. (2001) included five upper limb amputees with PLP and five without PLP in which the first complete muscle above the stump was used as a target for the TMS assessments. Although the number of patients was limited, there was an increase of the mapped area for the muscles in the amputated site and a medial displacement of the CoG only in patients with PLP. Also, the five patients with PLP presented increased cortical excitability (motor-evoked potentials were larger) when compared with the ones without PLP. These changes in cortical excitability are another important pattern of cortical reorganization frequently observed in patients that experience PLP. TMS studies in amputees showed increased excitability in the stump muscles (lower motor threshold and higher motor evoked potential amplitudes), and this response could be observed in a large scalp area than in the intact hemisphere (Cohen et al., 1991; Kew et al., 1994; Röricht et al., 1999). Mechanistic studies suggest that the increase in excitability after amputation is a result of the down-regulation of gamma-aminobutyric acid (GABA)-related inhibitory circuits (Ziemann et al., 1998). Further TMS evidence shows the increased motor cortex excitability by decreased intracortical inhibition paradigms measured by paired-pulse stimulation (Chen et al., 1998; Schwenkreis et al., 2000; Hordacre et al., 2015). Moreover, studies using neuroimaging techniques, such as fMRI and Positron emission tomography (PET), are in agreement with TMS data showing that BOLD activations in both somatosensory and motor cortices are significantly greater in patients with phantom limb pain (Kew et al., 1994).

Besides the alterations in brain activity observed in the contralateral area corresponding to the amputated limb, there is strong evidence of increased motor excitability in areas ipsilateral to the lost limb. For example, ischemic nerve block of the right-hand induced a transient increase in motor control in the left hemisphere; this process seems also to be driven by changes in GABA-ergic modulation (Werhahn et al., 2002). Although this review did not focus in changes in motor cortex excitability measured by the TMS, data suggests that the lack of inhibition in the motor cortex may be contributing to the underlying phantom limb pain mechanisms.

Furthermore, the amputees with PLP presented a medial somatosensory displacement of the mouth area into the hand area, similarly to the motor cortex displacement (Karl et al., 2001). This somatosensory reorganization was significantly correlated with the intensity of PLP. Therefore, the motor cortex reorganization might be secondary to somatosensory cortex changes. These results suggest that the motor cortex reorganization might be a better marker for the presence of PLP vs. amputation with no pain. However, larger studies are needed to gather better data and adequately test the hypothesis in which the deafferentation results in disrupted functional cortical representations and that this disruption is associated with the presence of pain.

### The Intensity of Pain Is Associated With the Level of Reorganization (Maladaptive or Abnormal/Enlargement), or It Is Related to the Level of Preservation of the Limb Representation

Although several studies documented an association between the presence and/or the intensity of PLP (and other types of chronic pain) with cortical reorganization alterations (shifts) in the representation of sensory and motor maps in humans, there is a current debate regarding the concept of reorganization. For example, fMRI studies showed that expansion or shift of the lip representation into the amputated hand area is correlated with higher pain levels: greater cortical remapping—more intense pain (Flor et al., 1995; Lotze et al., 2001; Raffin et al., 2016). Additionally, one study showed that after mirror therapy there is a reversal of this dysfunctional shift, which is significantly correlated with the reduction in phantom limb pain (Foell et al., 2014). However, it has been proposed that phantom pain in upper limb amputees is independent of cortical remapping, and it is associated with increased inputs into the cortical representation zone of the amputated limb (Makin et al., 2013). In this case, higher local activity and structural integrity lead to greater pain intensity. Some differences in techniques in the imaging analysis and experimental protocols could have been the reason for these mixed (but not mutually excluding) findings and should be considered when evaluating the literature. Even though TMS studies showed evidence of cortical reorganization following amputation and this reorganization is mainly observed in amputees with PLP, so far, the relationship between the intensity of pain with the amount of reorganization remains unclear. Our results suggest a lack of association between CoG shift and intensity of pain, but an association between these changes and the presence of pain—i.e., greater cortical remapping, the higher probability of having PLP. These observations suggest that deafferentation and alterations in local excitability patterns (decrease in inhibitory activity) showed by TMS and MRI studies can lead to shifts in network connections that can facilitate cortical reorganization that might lead to PLP. Moreover, the amount of reorganization—that could be a result of unmasking synaptic connection due to lack of inhibition—is associated with the intensity of pain as described by fMRI studies.

TMS assessment of cortical reorganization provides insights into PLP underlying mechanisms. Understanding the mechanisms of central reorganization can be used to explain the potential pain modulation effects of neuromodulation techniques, such as non-invasive brain stimulations (Collins et al., 2018; Meeker et al., 2019). Regarding the previous studies showing the top-down motor cortex modulation of pain networks through thalamocortical connections (Garcia-Larrea et al., 1999; Miranda et al., 2006; Yoon et al., 2014), the motor cortex can be a potential treatment target to decrease PLP. Therefore, the modulation of sensory-motor plasticity by non-invasive brain stimulation techniques, such as transcranial direct current stimulation, can play a role to optimize rehabilitation, and pain management of patients with amputation (Pinto et al., 2016).

However, the role of the somatosensory cortex and its reorganization after an amputation is far from being understood. Therefore, more studies evaluating larger samples and using more than one measurement of reorganization are necessary to elucidate this discussion. In the case of TMS studies, the main limitation is the numerous methodological variants observed across the performed studies as there is a wide variety of grid sizes, shape designs, and assessment protocols. Due to sample heterogeneity (upper or lower limbs amputation), several sizes of grids were observed as to account for the different sizes and position of the cortical representation of the evaluated limp or determined muscle. The variation of the prothesis use and type in studies can be another reason of the different results by affecting motor and sensory areas (Di Pino et al., 2014; Ferreri et al., 2014). The heterogeneity of trial design, population idiosyncrasy, and small sample sizes can also explain these mixed findings. The sample size among studies reviewed ranged from 1 to 15 patients, which may produce unpowered results and a more significant chance of a type II error.

## Conclusion

In conclusion, our review provides further evidence for post-amputation cortical reorganization in the affected motor cortex and suggests that the cortical reorganization is seen mainly in patients with PLP but not correlated with the intensity of PLP. Besides that, the stability and reliability of TMS measures across time support the use of TMS in studying cortical plasticity in amputees (Hetu et al., 2011). Given the limitations of the current data, longitudinal studies with larger sample size, and more homogeneous populations are needed to define underlying cortical mechanisms of PLP and their association with clinical parameters. The role of motor cortex reorganization is of high clinical interest, especially when applied for prevention and treatment response of phantom limb pain.

## Data Availability Statement

The raw data supporting the conclusions of this article will be made available by the authors, without undue reservation, to any qualified researcher.

## Author Contributions

MG is the first author and with FF conceptualized the paper. CP and MG wrote the Abstract. CP, FS, FL, and MG wrote the Introduction. KP-B and MG wrote the Methods and Results. MG, CP, FS, and DD wrote the Discussion. KP-B prepared the figure. FF provided critical review. All authors contributed to manuscript revision, read, and approved the submitted version.

### Conflict of Interest

The authors declare that the research was conducted in the absence of any commercial or financial relationships that could be construed as a potential conflict of interest.
