# Supplementary material for: Motor Cortex Reorganization in Limb Amputation: A Systematic Review of TMS Motor Mapping Studies
Source: Front Neurosci. 2020 Apr 21;14:314. doi: 10.3389/fnins.2020.00314 (PMC7187753; doi:10.3389/fnins.2020.00314)
Supplement: Supplementary file 1 [file Table_1.DOCX]

| **Supplementary Table S1**. Risk of Bias evaluation of included TMS studies | | | | | | | | | | | | | | | | | | | | | | | | | | | | |
| --- | --- | --- | --- | --- | --- | --- | --- | --- | --- | --- | --- | --- | --- | --- | --- | --- | --- | --- | --- | --- | --- | --- | --- | --- | --- | --- | --- | --- |
| **Studies** | **Age of subjects** | **Gender of subjects** | **Handedness of subjects** | **Subjects prescribed medication** | **Use of CNS active drugs** | **Presence of neurological/psychiatric disorders when studying healthy subjects** | **Any medical conditions** | **History of specific repetitive motor activity** | **Position and contact of EMG electrodes** | **Amount of relaxation/contraction of target muscles** | **Prior motor activity of the muscle to be tested** | **Level of relaxation of muscles other than those being tested** | **Coil type** | **Coil orientation** | **Direction of induced current in the brain** | **Coil location and stability (with or without a neuronavigation system)** | **Type of stimulator used** | **Stimulation intensity** | **Pulse shape** | **Determination of optimal hotspot** | **The time between MEP trials** | **Time between days of testing** | **Subject attention (level of arousal) during testing** | **Method for determining threshold (active/resting)** | **Number of MEP measures made** | **Method for determining MEP size during analysis** | **Size of unconditioned MEP** | **Score** |
| Schwenkreis (2003) | 1 | 1 | 1 | 0 | 0 | NA | 0 | 0 | 1 | 0 | 0 | 0 | 1 | 1 | 1 | 0 | 1 | 1 | 0 | 1 | 0 | 1 | 0 | 1 | 1 | 1 | 1 | 15 |
| Irlbacher (2002) | 1 | 1 | 1 | 0 | 0 | NA | 0 | 0 | 1 | 0 | 0 | 0 | 1 | 1 | 1 | 0 | 1 | 1 | 1 | 1 | 0 | 1 | 0 | 1 | 1 | 1 | 0 | 15 |
| Karl (2001) | 1 | 1 | 1 | 0 | 0 | NA | 0 | 0 | 1 | 0 | 0 | 0 | 1 | 1 | 1 | 0 | 1 | 1 | 0 | 1 | 1 | 1 | 0 | 1 | 1 | 1 | 1 | 16 |
| Schwenkreis (2001) | 1 | 1 | 1 | 0 | 0 | NA | 0 | 0 | 1 | 0 | 0 | 0 | 1 | 1 | 1 | 0 | 1 | 1 | 0 | 1 | 0 | 1 | 0 | 1 | 1 | 1 | 1 | 15 |
| Cohen (1991) | 1 | 1 | 0 | 0 | 0 | NA | 0 | 0 | 1 | 0 | 0 | 0 | 1 | 1 | 1 | 0 | 1 | 1 | 0 | 1 | 0 | 1 | 0 | 1 | 1 | 1 | 0 | 13 |
| Gagné (2011) | 1 | 1 | 1 | 1 | 1 | NA | 0 | 0 | 1 | 1 | 0 | 0 | 1 | 1 | 1 | 1 | 1 | 1 | 0 | 1 | 1 | 1 | 0 | 1 | 1 | 1 | 1 | 20 |
| Hamzei (2001) | 1 | 1 | 0 | 0 | 0 | NA | 0 | 0 | 1 | 0 | 0 | 0 | 1 | 0 | 1 | 0 | 1 | 1 | 0 | 1 | 1 | 1 | 0 | 1 | 1 | 1 | 1 | 14 |
| Kew (1994) | 1 | 1 | 1 | 0 | 0 | NA | 0 | 0 | 1 | 0 | 0 | 0 | 1 | 0 | 0 | 0 | 1 | 1 | 0 | 1 | 0 | 1 | 0 | 1 | 1 | 1 | 1 | 13 |
| Röricht (1999) | 1 | 1 | 0 | 0 | 0 | NA | 0 | 0 | 1 | 0 | 0 | 0 | 1 | 1 | 1 | 0 | 1 | 1 | 0 | 1 | 0 | 1 | 0 | 1 | 1 | 1 | 1 | 14 |
| Dettmers (1999) | 1 | 1 | 1 | 0 | 0 | NA | 1 | 0 | 1 | 1 | 0 | 0 | 1 | 0 | 1 | 0 | 1 | 1 | 0 | 1 | 0 | 1 | 0 | 1 | 1 | 1 | 1 | 16 |
| Pascual-Leone A (1996) | 1 | 1 | 1 | 1 | 1 | NA | 1 | 0 | 1 | 0 | 0 | 0 | 1 | 0 | 0 | 0 | 1 | 1 | 0 | 1 | 0 | 1 | 0 | 1 | 1 | 1 | 1 | 16 |
| Notes: 1=reported, 0=not reported, Score calculated out of 26 applicable domains. | | | | | | | | | | | | | | | | | | | | | | | | | | | | |
